# Application of Biotechnology in Specific Spoilage Organisms of Aquatic Products

**DOI:** 10.3389/fbioe.2022.895283

**Published:** 2022-04-28

**Authors:** Huina Dong, Yuanming Gai, Shaoping Fu, Dawei Zhang

**Affiliations:** ^1^ Tianjin Institute of Industrial Biotechnology, Chinese Academy of Sciences, Tianjin, China; ^2^ University of Chinese Academy of Sciences, Beijing, China

**Keywords:** specific spoilage organisms, aquatic products, spoilage indicator, quorum sensing, preservation strategies

## Abstract

Aquatic products are delicious and have high nutritive value, however, they are highly perishable during storage due to the growth and metabolism of microorganisms. The spoilage process of aquatic products was demonstrated to be highly related to the composition of microorganisms, in which the specific spoilage organisms (SSOs) are the main factors. In this article, the spoilage indicators of SSOs were systematically described, which could make a comprehensive evaluation of the quality of aquatic products. Quorum sensing (QS) regulates the growth, metabolism and characteristics of SSOs, the common signaling molecules and the QS system in the major SSOs of aquatic products were discussed. Moreover, we compared various technologies for the analysis of SSOs in aquatic products. Besides, quality control techniques based on microbiota regulating of aquatic products, including physical, chemical and biological preservation strategies, were also compared. In conclusion, novel preservation technologies and hurdle techniques are expected to achieve comprehensive inhibition of SSOs.

## 1 Introduction

Aquatic products are rich in protein, fat, vitamins, and minerals, and are very popular because of their delicacy and high nutritive value. However, the high contents of various nutrients and moisture of aquatic products limited their shelf-life (Gudipati 2017). The changes in sensory and nutritional properties resulted from the rapid microbial growth and metabolism and biochemical reactions occur in aquatic products after death (Olatunde and Benjakul 2018). The composition of microorganisms especially bacteria is also associated with spoilage process and spoilage profiles of aquatic products (Duan et al., 2018; Parlapani et al., 2020). Microbiota in aquatic products alters dramatically along with the storage time and according to many other factors such as aquaculture species and environment, processing operation, preservation conditions and some quality control techniques (Hauptmann et al., 2020).

At early period of storage of aquatic products, microbiota composition undergoes dramatic changes usually reflected in a decrease in microbial community richness and diversity (Zhang et al., 2019; Zhuang et al., 2019). As the storage time increases, only a few kinds of bacteria will become dominant and ultimately lead to the spoilage of aquatic products (Zhuang et al., 2019). These bacteria were called specific spoilage organisms (SSOs) because of their major roles in the spoilage process of aquatic products. Microbial community remains relatively stable after the sensory rejection point, although the relative abundance of different SSOs might still vary slightly.

The study on the characteristics of SSOs is important for the quality control techniques used for aquatic products preservation. Therefore, this paper systematically described the spoilage indicators of SSOs, including volatile compounds (biogenic amines, trimethylamine and total volatile base nitrogen), thiobarbituric acid and *K*-value, which could make a comprehensive evaluation of the quality of aquatic products. The common signaling molecules and the QS system in the major SSOs of aquatic products were also been discussed. Moreover, we compared various analysis technologies present used in microbiota characterization in aquatic products. Besides, preservation technologies based on regulating microbial communities of aquatic products were also concluded.

## 2 The Spoilage Indicators of SSOs

There are significant differences in the spoilage potentials of different SSOs. Sensory score and total viable counts are traditional and helpful indicators to assess the freshness of aquatic products. The spoilage potentials of SSOs were positively correlated with sensory score and total viable counts. However, only rely on the sensory score and total viable counts are insufficient to directly say SSOs influence on the product. Many metabolites (such as volatile compounds etc.) are produced by the SSOs of aquatic products, which can reflect the spoilage potentials of SSOs and the freshness of aquatic products. These metabolites of SSOs can be used as indicators to make a comprehensive evaluation of the quality of aquatic products. The variation trend of each index has a high linear correlation, which is of great significance to understand the characteristics of different SSOs or the spoilage characteristics of different aquatic products. These indicators mainly include volatile compounds (biogenic amines, trimethylamine and total volatile base nitrogen), thiobarbituric acid and K value.

### 2.1 Volatile Compounds

#### 2.1.1 Biogenic Amines

**FIGURE 1:**
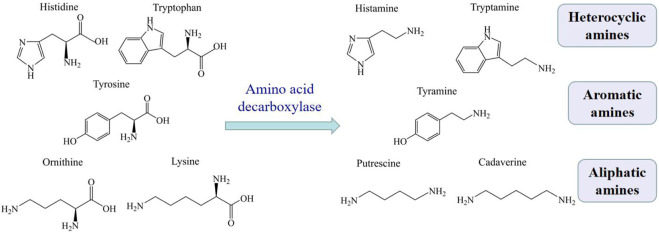
The production of major BAs in aquatic products.

Biogenic amines (BAs) are basic nitrogenous compounds with low molecular weight, which are formed by decarboxylation of free amino acids under the action of microbial decarboxylase, or amination and transamination of aldehydes or ketones (Brink et al., 1990). The major BAs in aquatic products are histamine, tyramine, tryptamine, putrescine, and cadaverine, which are formed by removing the α-carboxyl group in their respective free amino acids (see Figure 1) (Biji et al., 2016). According to the chemical structure, these BAs can classify into heterocyclic amines (histamine and tryptamine), aromatic amines (tyramine) or aliphatic amines (putrescine and cadaverine).

Histamine is the most important and thoroughly studied amine among all the BAs (Biji et al., 2016). Many bacterial species could produce histamine, including *Pseudomonas putida*, *Pseudomonas fluorescens*, *Aeromonas* spp. (Hwang et al., 2010). Histamine and tyramine are considered as anti-nutrients, and consuming too much of them can result in histamine poisoning and tyramine toxicity (Chong et al., 2011). Putrescine and cadaverine sometimes react with nitrite to form carcinogenic nitrosoamines, although they do not have any adverse health effect (Onal et al., 2013).

The levels of BAs can be considered as a spoilage indices as the BAs usually were produced at the end of shelf-life (Özogul and Özogul 2006). The biogenic amine index (BAI) is the sum of the content of histamine, tyramine, putrescine and cadaverine (Biji et al., 2016). When BAI of tuna is < 50 mg/kg, it indicates the tuna has acceptable quality, whereas BAI >45 and >90 mg/kg indicate initial and advanced tuna decomposition, respectively (Du et al., 2002). The acceptable limit of BAI of anchovy and barramundi is only 15–16 mg/kg (Pons-Sánchez-Cascado et al., 2006; Bakar et al., 2010).

#### 2.1.2 Trimethylamine

Trimethylamine (Hauptmann, Paulova *et al.*), as a precursor of carcinogen nitrosamine, is an important smelly odor and is usually used as an indicator of aquatic freshness and quantitative indicator of SSOs metabolites. TMA could be formed from the trimethylamine oxide by bacterial enzyme activity (Sotelo et al., 1995).

TMA content was affected by different temperature and storage methods. TMA content at 10°C was much higher than that at 4°C, which resulted in the end point of spoilage reached much earlier (Parlapani et al., 2019). However, the changes in TMA content cannot comprehensively evaluate the spoilage degree of aquatic products and was gradually replaced by total volatile base nitrogen level.

#### 2.1.3 Total Volatile Base Nitrogen

Total volatile base nitrogen (TVB-N) refers to the decomposition of proteins in aquatic products under the interaction of microorganisms and enzymes to generate low basic volatile nitrogen-containing substances such as ammonia and amines. TVB-N content has a certain correlation with the spoilage degree of aquatic products, which is one of the most widely used indicators to measure the freshness of aquatic products. TVB-N was positively correlated with the total number of bacteria, which could effectively reflect the number of SSOs bacteria and the quality of aquatic products (Gui et al., 2018). TVB-N values of whole ungutted sea bass during storage had slightly increased and reached 26.77 mg N per 100 g muscle at day 13, which regarded as the limit of acceptability, whereas TVB-N values of filleted fish reached the limit value only at day 9 (Taliadourou et al., 2003). The acceptable limit of TVB-N level of European eel is about 10 mg TVB-N per 100 g flesh (Özogul et al., 2005).

The quantitative index of bacterial spoilage ability, the yield factor of spoilage metabolites (Y_TVB-N/CFU_), is the quantity of spoilage metabolites produced by unit spoilage bacteria at the end point of spoilage. Taking the value of Y_TVB-N/CFU_ as the quantitative standard of SSOs spoilage ability can well reflect the degree of spoilage of aquatic products. The higher the value of Y_TVB-N/CFU_ is, the stronger the SSOs decaying ability is. Y_TVB-N/CFU_ values of *Pseudomonas* spp. *Acinetobacter* spp. and *Brochothrix thermosphacta* isolated from chilled raw tuna (*Thunnus obesus*) were tested, and the results shown that *Pseudomonas* spp. played the most important role in spoilage process (Liu et al., 2018). Higher Y_TVB-N/CFU_ value of *P. fluorescens* on salmon was observed in samples stored at lower temperatures than at high temperature (Xie et al., 2018).

### 2.2 Thiobarbituric Acid

Aquatic products is rich in unsaturated fatty acids (Rodriguez-Casado et al., 2007), which can be easily oxidized and gradually break down into the low molecular weight substances such as aldehydes, ketones and carboxylic acid groups. These substances will change the smell, texture, color and nutritional values of aquatic products (Trocino et al., 2012). Lipid oxidation is in relation to lipase activity of SSOs. Thiobarbituric acid (TBA) value is a helpful indicator to predict the level of lipid oxidation and assess aquatic products freshness. TBA content is usually expressed as mg malonaldehyde (MDA)/kg muscle. *P. fluorescens* TBA content increased with the extension of storage time in all samples, and TBA values of filleted sea bass were significantly higher than that of whole ungutted sea bass samples, indicating that the degree of oxidative rancidity in filleted fish was higher (Taliadourou et al., 2003).

### 2.3 *K*-Value

**FIGURE 2:**
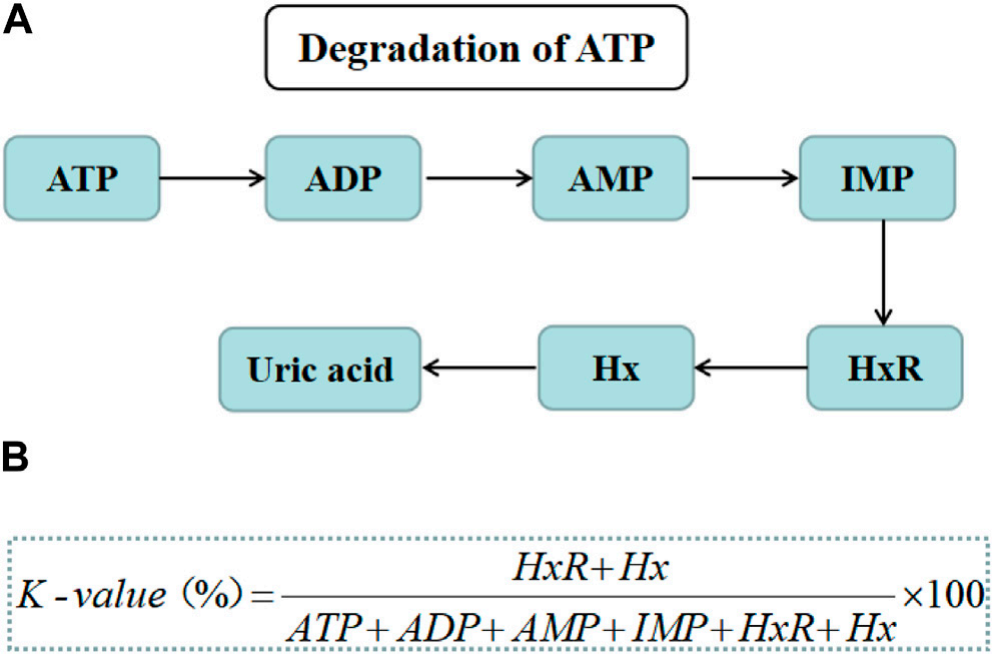
**(A)** The ATP degradation process. **(B)** The formulas used for calculating *K*-value.

The decomposition of adenosine triphosphate (ATP) is predominant in the elaborate postmortem changes of aquatic products. The ATP breakdown process including from ATP to adenosine diphosphate (ADP), adenosine monophosphate (AMP), inosine monophosphate (IMP), and inosine (HxR), can reflect the first changes in aquatic products before bacterial growth (Figure 2A). However, the production of hypoxanthine (Hx) from HxR can be favored by SSOs activity, such as Shewanella putrefacien (Jia et al., 2019). *K*-value was defined as the ratio (%) of the total amount of HxR and Hx to that of ATP-related compounds (Figure 2B) (Cheng et al., 2015).

A higher *K*-value indicates a higher ATP decomposition rate. *K*-value is also an useful indicator to evaluate the freshness of aquatic products (Wills et al., 2004). The *K*-value of filleted ray fish showed an exponential increase during storage, indicating the signs of freshness and deterioration (Ocaño-Higuera et al., 2011). The *K*-value of tray-packed tilapia fillets was highly correlated with storage time and sensory acceptability (Shouchun et al., 2010).

## 3 Quorum Sensing Systems in SSOs

Bacterial quorum-sensing (QS) is a regulatory system in which bacteria sense environmental changes by using extracellular signaling molecules and activate related gene expression to adapt to environmental changes (Fuqua et al., 1994). In QS, the communication route is considered to be a cell density-dependent signal transduction phenomenon and is involved in many important biological processes, such as sporulation, virulence and pathogenesis, food spoilage, biofilm formation (Machado et al., 2020), synthesis of antibiotics (McCormack 2006), combined transfer of plasmids (Zhang et al., 1993) and symbiosis between rhizobia and plants (Reading and Sperandio 2006).

**FIGURE 3:**
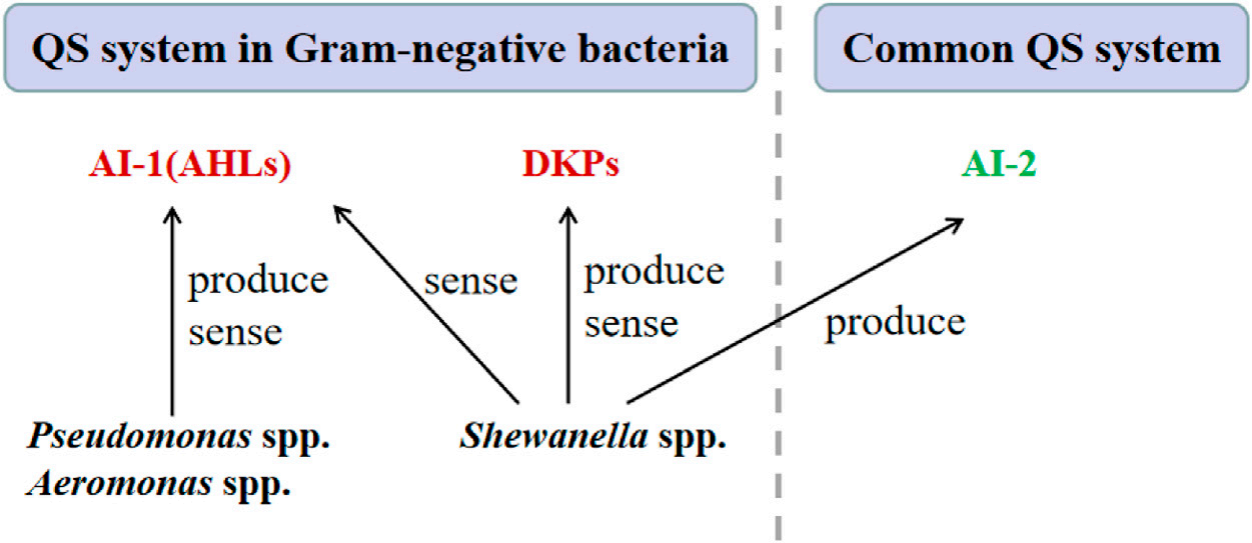
Schematic representation of the QS mechanisms of the major SSOs in aquatic products.

The major spoilage bacteria in aquatic products including *Pseudomonas* spp. *Aeromonas* spp. and *Shewanella* spp. The signaling molecules of these bacteria and the corresponding QS system will be introduced in the fallowing, and their interrelationship is shown in Figure 3.

### 3.1 Signaling Molecules

Several types of signaling molecules, also known as autoinducers, have been discovered in the microbiota of aquatic products, including N-acyl-homoserine lactones (AHLs), autoinducer-2 (AI-2) and diketopiperazines (DKPs).

**FIGURE4:**
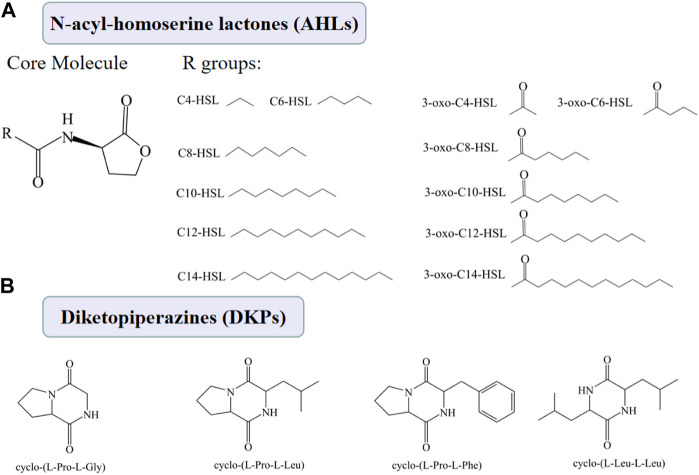
Chemical structures of AHLs **(A)** and DKPs **(B)**.

AHLs is the autoinducer of the LuxI/LuxR-type QS mediated QS system, which is the major QS system in Gram-negative bacteria (Machado et al., 2020). The AHLs are synthesized by an AHL synthase (LuxI), and an AHL receptor (LuxR type family transcription regulator). Although there are many types of AHLs (Figure 4A), all AHLs contain a conserved acylated homoserine lactone (HSL). The mechanisms by which AHLs regulate gene expression are similar, so are the regulatory mechanisms of different species of Gram-negative bacteria.

AI-2 is the autoinducer of a common QS system that can be found in almost one-half of bacterial genomes and is considered the most ubiquitous signaling system employed by both Gram-negative and Gram-positive bacteria (Abisado et al., 2018). AI-2 is synthesized by the LuxS enzyme from 5-dihydroxy-2,3- pentanedione (DPD), and is transconducted to the transcriptional regulator (e.g., LuxR and LsrR) over membrane by the LuxPQ proteins (Waters and Bassler 2005).

DKPs, shown in Figure 4B, have been considered as another type of autoinducer involved in the QS system of some Gram-negative bacteria, such as *P. putida* and *Shewanella baltica* (Gu et al., 2013). However, the specific mechanism of DKPs-mediated QS system has not been fully elucidated.

### 3.2 QS System of *Pseudomonas* spp.

The QS system of *Pseudomonas* is mainly related to AHLs. Three types of AHLs, with C4-HSL was a major one, were detected in the extract of *P. fluorescens* isolated from spoiled large yellow croaker (Tang et al., 2019). A LuxI/LuxR homolog was identified in this strain and the C4-HSL was almost undetectable in the in-frame deletion mutant of luxI and decreased greatly in the mutant of luxR. P. fluorescens mutants showed significant decreases in biofilm biomass and exopolysaccharide production, and apparent attenuates in spoilage factors, siderophore and protease. The C4-HSL produced by *Pseudomonas* psychrophila PSPF19 isolated from freshwater fish induced exoenzyme production and increased attachment and biofilm formation (Bai and Rai Vittal 2014).

Except for C4-HSL, six other AHLs (3-oxo-C12-HSL, 3-oxo-C14-HSL, 3-oxo-C6-HSL, 3-oxo-C8-HSL, C12-HSL, C6-HSL) were detected in the culture of *P. psychrophila* KM0 and *P. fluorescens* isolated from salmon and 3-oxo-C12-HSL and C4-HSL were the major AHLs (Sobieszczanska et al., 2020).

### 3.3 QS System of *Aeromonas* spp.

The QS system of *Aeromon*as spp. is also mainly related to AHLs. *Aeromonas* sobria AS7 from turbot samples could produce five types of AHLs, including C4–HSL, C6–HSL, C8–HSL, C10–HSL and C12–HSL, among which C4–HSL and C8–HSL are the most important AHLs in particular (Li et al., 2016). Exogenous C8–HSL regulated siderophore production, while exogenous C4–HSL and C8–HSL both could accelerate the growth rate and population density of A. sobria AS7. *Aeromonas* veronii LP-11 from sturgeon produced C6-SHL, C8-HSL, 3-oxo-C8-HSL and 3-OH-C8-HSL, and the QS system may have been involved in the regulation of sturgeon spoilage (Gui et al., 2018).

The same five types of AHLs as A. sobria AS7 were detected in the extract of *Aeromonas* salmonicida isolated from large yellow croaker, and C4-HSL was a major signal molecule (Li Liu et al., 2018). AsaI/C4-HSL played an important role in spoilage, motility and biofilm formation of A. salmonicida. A. salmonicida strain Keldur 265–87 isolated from diseased fish only synthesized C4-HSL (Schwenteit et al., 2011).

### 3.4 QS System of *Shewanella* spp.

Although *Pseudomonas* spp. and *Aeromonas* spp. are mainly to produce AHLs, *Shewanella* spp. may not produce AHLs as no related genes were found in genomes of Shewanella spp. and no AHLs were detected in the culture of Shewanella spp. (Zhu et al., 2015). However, Shewanella spp. can sense some AHLs which could regulate the production of biofilm matrixes and extracellular proteases, and thus change their spoilage capabilities (Zhu et al., 2015).

S. baltica from large yellow croaker could produce four kinds of DKPs, including cyclo-(l-Pro-l-Gly), cyclo-(l-Pro-l-Leu), cyclo-(l-Leu-l-Leu), and cyclo-(l-Pro-l-Phe) (Gu et al., 2013). These four DKPs could correspondingly enhance the spoilage capability of S. baltica and cyclo-(L-Pro-L-Phe) had the greatest promoting effect. These four DKPs also could regulate biofilm formation and three potential critical spoilage gene expression of *S. baltica* (i.e., *torT*, *cysM* and *trxB*) (Fu et al., 2018). The other two studies found *S. baltica* isolated from large yellow croaker could only produce cyclo-(l-Pro-l-Leu) and cyclo-(l-Pro-l-Phe) and without cyclo-(l-Pro-l-Gly) and cyclo-(l-Leu-l-Leu) (Zhu et al., 2016; Zhu et al., 2019).

*S. balti*ca and S. putrefaciens also could produce AI-2 (Zhu et al., 2015), however, they could not sense AI-2. The luxS deficient mutant of S. baltica demonstrated AI-2 might not play a signaling role in spoilage (Zhu et al., 2016).

## 4 The Analysis Technologies of SSOs

Traditional SSOs analysis methods include microscopic examination, plate culture, biochemical reaction, gas production experiment, etc., but these methods have a long cycle, low sensitivity and accuracy, and not all microorganisms in aquatic products can be cultured. With the development of molecular biotechnology, molecular diagnostic technology is widely used in SSO analysis, and polymerase chain reaction (PCR) is widely used in the field of food microbiology. The analysis technologies of SSOs in aquatic products mainly include repetitive-element PCR (Rep-PCR), PCR denaturing gradient gel electrophoresis analysis (PCR-DGGE), PCR restriction fragment length polymorphism technology (PCR-RFLP) and high-throughput sequencing.

### 4.1 The Analysis Technology of Rep-PCR

Rep-PCR is a DNA sequence-based typing technique. It is based on PCR amplification using targeted primers of widely distributed conserved, interspersed repetitive DNA elements in bacterial chromosomes. These repetitive DNA elements have differences in their distribution and copy number at the level of strain, species and genus in the bacterial genome, and the sequence itself is highly conserved in the evolutionary process (Bloch and Rode 1996). By comparing the electrophoresis results of PCR products, we can analyze the differences between the genomes of bacterial strains.

More than 10 short repeats of bacterial genome repeats have been identified in the genome of bacteria, and the most widely reported are Repetitive Extragenic Palindrome (REP) (Bloch and Rode 1996). The REP-PCR was used to genetically characterize the *Vibrio parahaemolyticus* isolates from 150 aquatic products samples (Paydar et al., 2013). The results showed that 41 REP profiles could be observed and all isolates were classified into 11 different clusters with 80% similarity. The DNA finger map of *V. para*haemolyticus showed a high degree of genetic diversity among isolates, and REP-PCR can distinguish isolates with different virus types (Ma et al., 2013). REP-PCR was used to screen 158 Edwardsiella piscicida isolates recovered from diseased channel and hybrid catfish in Mississippi to assess intraspecific genetic variability (López-Porras et al., 2021). The operation of this method is relatively simple and can provide some valuable reference for isolating SSOs of aquatic products.

### 4.2 The Analysis Technology of PCR-DGGE

16S rDNA is the most widely used molecular clock for bacterial systematic classification. Its evolution has a good clock nature and a high degree of conservation in structure and function. The sequence of 16S rDNA contains nine variable regions and 10 constant regions (Cao et al., 2017). The conserved sequence region reflects the genetic relationship between species, while the highly variable sequence region reflects the differences between species. The sequence characteristics of 16S rDNA molecules laid a molecular basis for the systematic classification of related species at different classification levels. 16S rDNA amplicon sequencing technology uses the conserved region sequence to design primers, amplifies the highly variable region sequence and performs sequencing identification, which plays an important role in the study of microbial community composition.

DGGE uses gel electrophoresis to determine the amplification products of bacterial 16S rDNA fragments based on the specificity and stability of DNA structure. The separation of 16S rDNA fragments is based on the reduced electrophoretic mobility of partially melted double stranded DNA molecules in polyacrylamide gels with a linear gradient of denaturing agents, such as urea or formamide (Flórez and Mayo 2006). The adding of denaturing agents can separate the DNA fragments of the same size but with different bases, as the DNA fragments of different bacteria have their specific DNA unwinding regions and unwinding rules (Fischer and Lerman 1983). A GC clamp of approximately 50 bp is attached to the 5’ end of one of the primers to prevent complete disassociation of the two DNA strands (Sheffield et al., 1989).

PCR-DGGE technology can analyze the changes of microbial community composition and number in different samples under different conditions, and determine the dominant bacteria in the samples (Muyzer et al., 1993). Compared with other analytical techniques, PCR-DGGE technology has significant advantages in the detection of microbial community diversity and population difference. In recent years, PCR-DGGE technology has been increasingly applied to the study of SSOs in aquatic products. For example, PCR-DGGE was used to characterize the dominant bacterial population in fish, including Atlantic mackerel (Svanevik and Lunestad 2011), Atlantic salmon (Hovda et al., 2012), cod (Hovda et al., 2007a), farmed halibut (Hovda et al., 2007b) and tuna (Ge et al., 2012), and in Norway lobster (Bekaert et al., 2015) by amplification of the hypervariable V3 region on 16S rDNA.

### 4.3 The Analysis Technology of PCR-RFLP

RFLP technology is to detect the size of specific DNA fragments formed after restriction endonuclease digestion. The specificity of restriction endonuclease inhibits effective cleavage of microbial genomic DNA fragments. However, PCR and RFLP can be combined to obtain simple and effective molecular map, which is called PCR-RFLP. Ribosomal RNA genes are specific targets, among which 16S rDNA PCR-RFLP is the most simple and mature techniques and has been widely used.

A protocol based on the RFLP patterns of the complete PCR-amplified 16S rDNA can simultaneously identified most (10 species) of *Aeromonas* spp. using endonucleases *Alu*I and *Mbo*I (Borrell et al., 1997). However, the identification of *A*. *salmonicida*, *A*. *encheleia* and *Aeromonas* HG11 needed two additional enzymes, *Nar*I and *Hae*III. The protocol had been extended with endonucleases *Alw*NI and *Pst*I to separate the species of *A. salmonicida*, *A. bestiarum* and *A. popoffii* (Figueras et al., 2000). Another 16S rDNA PCR-RFLP analysis using the restriction enzymes (*Alu*I, *Mbo*I, *Pvu*II, *Pst*I and *Nar*I) was used to rapidly identify the *Aeromonas* genospecies isolated from diseased fish and aquatic animals (Rahman et al., 2005). SSO of grass carp during chilled storage were analyzed by 16S rDNA PCR-RFLP using endonuclease *Msp*I, the results shown that *Aeromonas* spp. was the predominant SSO (Pan et al., 2013).

### 4.4 High-Throughput Sequencing Technology

High-throughput sequencing (HTS) technology is a milestone in the development of DNA sequencing, mainly including the second generation of high-throughput sequencing technology represented by 454 (GS-FLX), Solexa Genome Analyzer and SOLiD, single-molecule sequencing technology represented by HeliScope TIRM and Pacific Biosciences SMRT, and Ion Personal Genome Machine (PGM) sequencing technology introduced by Life Science (Di Bella et al., 2013). HTS technology has many advantages, such as precise sequencing, high output and low cost, and has been widely applied in the research of SSOs of aquatic products. HTS has been successfully used to characterize the SSOs of various aquatic products, such as cod and salmon fillets (Chaillou et al., 2015), cold-smoked salmon (Leroi et al., 2015), oysters (Rong et al., 2018), and Spanish mackerel (Zheng et al., 2020).

## 5 Application of SSOs in Aquatic Products Preservation

**FIGURE 5:**
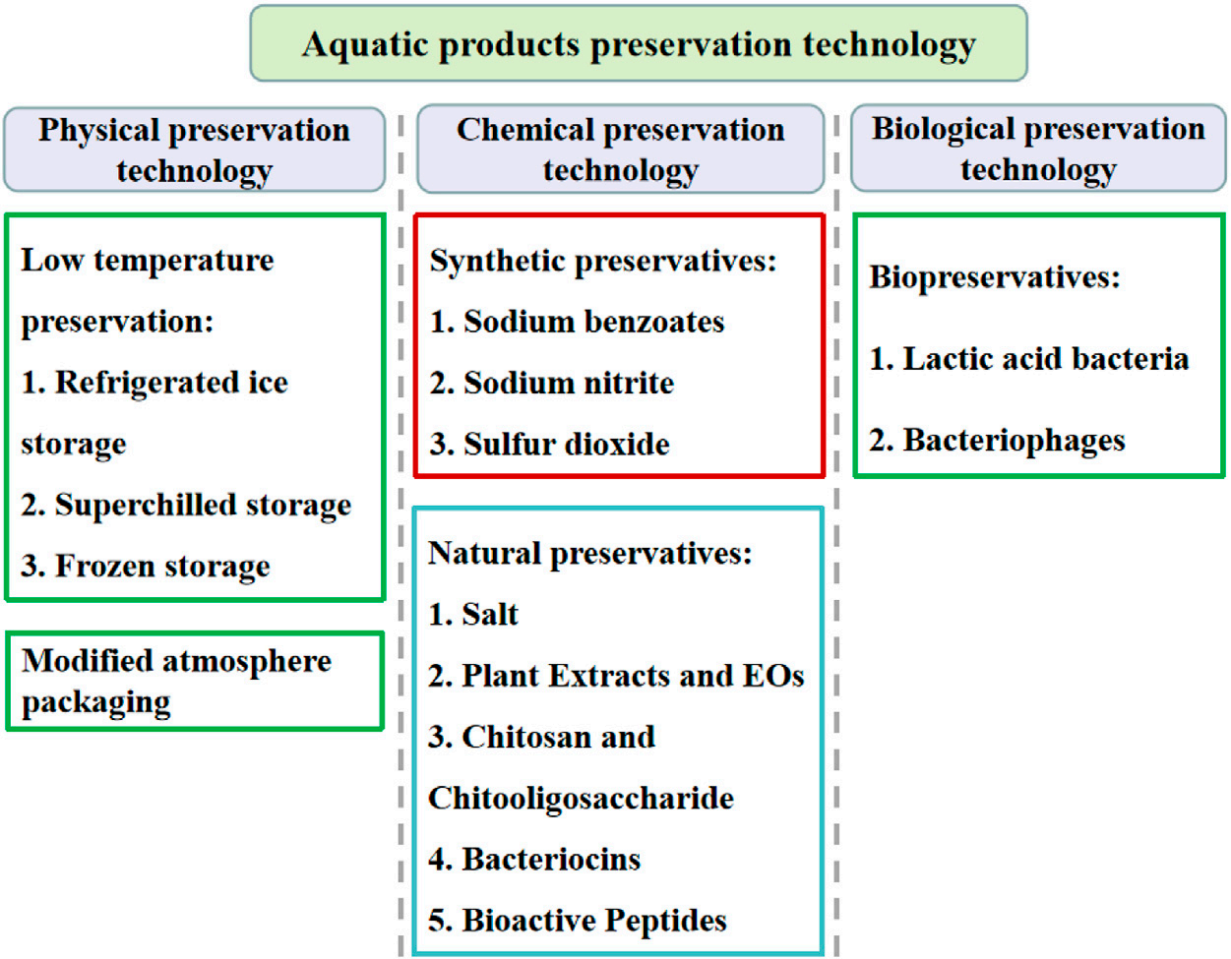
Classification of the aquatic products preservation technologies.

At present, the aquatic products preservation technologies used to control SSOs are mainly divided into physical, chemical and biological preservation strategies (Figure 5).

### 5.1 Physical Preservation Technology

#### 5.1.1 Modified Atmosphere Packaging

Modified atmosphere packaging (MAP) is a fresh-keeping method which can prolong the shelf life by adjusting the proportion and composition of air in aquatic product package. MAP mainly inhibits the growth and reproduction of spoilage microorganisms through CO_2_ (DeWitt and Oliveira 2016). And higher CO_2_ contents in packaging atmosphere more effectively inhibit bacterial growth and retard biochemical changes (Yew et al., 2014; Olatunde et al., 2020). MAP is often used in combination with other preservation strategies to extend shelf life of aquatic products such as low temperature or plant extracts (Navarro-Segura et al., 2020).

#### 5.1.2 Low Temperature Preservation Technology

Low temperature preservation technology is the most studied and widely used preservation technology of aquatic products. At present, the low temperature cold chain storage and transportation used in the world generally include refrigerated ice storage between 0 and 4°C, superchilled storage at -1 to −4°C, and frozen storage in the range of −18 to (−40)°C (Gallart-Jornet et al., 2007). Low temperature preservation technology can keep the freshness and nutritional quality of aquatic products by inhibiting the activities of microorganisms and enzymes.

Refrigerated ice storage is a method of keeping fresh aquatic products fresh by putting them into ice or cold sea water. Refrigerated ice storage is the most historical and traditional method of preservation widely used in the world. As refrigerated ice storage products are closest to the biological characteristics of fresh aquatic products, this method is still used today. Refrigerated ice storage extends shelf-life by reducing microbial reproduction rate at low temperature not completely inhibited, mainly used for short-term and small fish preservation, as the heat transfer inside the product is very slow. For large fish, the cooling time of this preservation method is too long, which is not conducive to the preservation. For example, the weight of 4–5 kg of salmon, with −1°C cold sea water cooling, the central temperature from 15 to 2°C need 2 h (Magnussen et al., 2008).

Superchilled storage is a method to store aquatic products in the temperature zone between 1 and 2°C below the initial freezing point. The aquatic products in the ice temperature zone can maintain the living nature (the state of death and dormancy), and reduce the speed of metabolism, so as to preserve the original color, aroma, taste and taste for a long time. It can also effectively inhibit the growth and reproduction of microorganisms, and inhibit the chemical changes such as lipid oxidation and non-enzymatic Browning in the food. Compared with refrigerated preservation, superchilled storage can keep freshness and nutrition of aquatic products better and prolong shelf life by 1.4–2.0 times (Que et al., 2013). And protein denaturation and other texture deterioration caused by freezing can be avoided when compared with frozen storage. Fernandez *et al.* (Fernández et al., 2009) applied three fresh-keeping methods of natural additives, superchilling, and MAP to extend the shelf-life of Atlantic Salmon fillets, and found that the shelf-life of salmon could be increased from 11 to 22 days by the combination of -1.5°C ice temperature and air conditioning. The superchilled modified atmosphere (MA) (CO_2_:N_2_ 60:40) packaged salmon fillets maintained a good quality, with negligible microbial growth for more than 24 days, whereas MA-stored fillets at chilled conditions was spoiled after only 10 days (Sivertsvik et al., 2006).

Frozen storage is a method of keeping aquatic products fresh by lowering the central temperature to −15°C and then storing and circulating them below −18°C. Because most of the water in the fresh water product tissue is frozen, the activity of microorganism and enzyme is inhibited, so that the shelf-life can be extended for several months and it is widely used in the market. However, protein denaturation can be easily caused by long-term frozen storage, and the sensory and nutritional quality of aquatic products will be decreased. The rapid freezing technology combined with frozen liquid can significantly reduce the impact on the quality of aquatic products and become the main development direction of this technology. The effects of −2.5°C storage and −20°C storage on physicochemical and sensory indexes of Snakehead fillets were studied (Que et al., 2015). The results showed that although the shelf-life could be significantly prolonged by frozen storage, the fish quality and the integrity of tissue structure could be better maintained by superchilled storage during a short storage period.

### 5.2 Chemical Preservation Technology

Chemical preservation technology is a method of keeping fresh by adding various drugs into aquatic products by virtue of their bactericidal or bacteriostatic effects. The most used chemical preservatives including sodium benzoates, sodium nitrite, and sulfur dioxide. However, these synthetic preservatives have potential harm to human health and are often not accepted by consumers (Olatunde and Benjakul 2018). Salt is the oldest and most commonly used natural preservative for extending the shelf-life of aquatic products as its low cost and simplicity (Martínez-Alvarez and Gómez-Guillén 2013). Salt storage is using the osmotic dehydration of salt solution to reduce the water content of fish body, and extending the shelf-life by destroying the microorganism and enzyme activity of aquatic products muscle. Nevertheless, uncontrolled growth of halotolerant and halophilic bacteria in salt-preserved aquatic products can also lead to products spoilage (Pikuta et al., 2007). Moreover, salting of aquatic products will affect the taste and result in high sodium content in aquatic products (Ormanci and Colakoglu 2015).

Other representative natural preservatives that can effectively replace synthetic preservatives or chemicals because of their excellent antimicrobial and antioxidant properties including plant extracts, essential oils (EOs), chitosan, bacteriocins, and bioactive peptides.

#### 5.2.1 Plant Extracts and Essential Oils

Plant extracts and EOs are natural preservatives derived from plant and widely used in the food industry (Chouliara et al., 2007). Plant extracts and EOs have abundant of phenolic compounds, which was considered to be responsible for their excellent antioxidant and antimicrobial activities (Olatunde and Benjakul 2018). The phenolic compounds can be divided into two categories, flavonoids and non-flavonoid polyphenols, among which the former one is the target class (Maqsood et al., 2014).

Phenolic compounds will increase the permeability of cell membrane, which ultimately result in cell death (Simoes et al., 2009). Plant extracts or EOs have a better inhibitory effect on Gram-negative bacteria possessing a thinner cell wall than Gram-positive bacteria (Abdollahzadeh et al., 2014). Zhuang *et al* (2021) reviewed the effects of various kinds of plant extracts or EOs on inhibiting spoilage bacteria, changing microbial composition, and prolonging shelf-life of fishery products, when used alone or in combination with biopolymer matrix.

However, a higher amount or concentration of plant extracts or EOs will be needed when they were applied in aquatic products than synthetic preservatives, which will result in the appearance and color not acceptable (García-Díez et al., 2016). Especially the EOs which are mostly extracted from herbs and spices have a strong aroma even at low concentration, resulting in negative effects on the aroma and taste of the treated aquatic products (Silva-Angulo et al., 2015). And the compounds variation in plants limited the applications of their extracts as natural preservatives in aquatic products (Weerakkody et al., 2010).

#### 5.2.2 Chitosan and Chitooligosaccharide

Chitosan is the product of deacetylation of chitin, which is the second most abundant natural polymer, while chitooligosaccharide is the product of depolymerization of chitosan (Lodhi et al., 2014). Chitosan and chitooligosaccharide both have amine, acetylated amine groups, and hydroxyl group, which can interact with cell receptors and trigger a series of reactions in living organisms. These structure characteristics are the major factor responsible for their antioxidant and antimicrobial properties (Lodhi et al., 2014). As their nontoxic, degradable, and natural attributes, they had been widely used in food industry (Alishahi and Aïder 2011). However, chitooligosaccharide was rarely reported to be used for extending the shelf-life of aquatic products.

Chitosan solution is widely used as an outer coating to extend shelf-life of aquatic products due to its film-forming ability. The shelf-life of brown trout were extended to 9 and 12 days for samples coated with chitosan dissolved in 1.5% lactic acid and 1.5% acetic acid, respectively, while the shelf-life of the control only has 6 days (Alak 2012). The eating quality of 1 and 2% chitosan-treated Indian oil sardine fillets in iced condition were maintained for up to 8 and 10 days respectively, compared to only 5 days for untreated samples (Mohan et al., 2012).

Chitosan was used in combination with other preservatives or treatments to enhance its preservative effect. Deep-water pink shrimp coated with chitosan film in the presence of 0.5–2.0% orange peel EO had an extended shelf-life (15 days) when compared to shrimp coated with chitosan only (10 days) (Alparslan and Baygar 2017). Oyster treated with ozone and coated with 2% chitosan had an extended shelf-life (20–21 days), compared with 8–9 days for the untreated sample (Rong et al., 2010). Chitosan is always dissolved in acidic solution, which may cause protein precipitation, loss in water-holding capacity, or a sour taste. The compounds such as fat, protein in aquatic products may interact with chitosan or chitooligosaccharide, which will lead to the loss of their antioxidant and antimicrobial properties (No, Meyers et al., 2007).

#### 5.2.3 Bacteriocins

Bacteriocins are proteins or polypeptides produced by some Gram-negative and Gram-positive bacteria (Zacharof and Lovitt 2012). Bacteriocins exhibit promising antimicrobial properties against a variety of pathogens and spoilage bacteria by mechanisms specific to each type and bacteriocin (Perez et al., 2015). In general, the interaction between bacteriocins and cell membrane of the target strain play an important role in antimicrobial action of bacteriocins. However, bacteriocins do not reduce or prevent lipid oxidation (Olatunde and Benjakul 2018).

Bacteriocins were used to inhibit the growth of pathogenic and spoilage bacteria in fish. Lactic acid bacteria (LAB) could produce a number of bacteriocins, which are reported to possess profound bactericidal potency against food spoilage microorganisms, such as *Bacillus cereus*, *Staphylococcus aureus*, and *Pseudomonas aeruginosa* (Bali, Panesar et al., 2016). Bacteriocins produced by *Bacillus* sp. exhibited strong antimicrobial activity against *Salmonel*la spp. and *Vibrio* spp. isolated from marine fish and squid (Ashwitha et al., 2017).

Bacteriocins combined with other preservatives or methods will result in increased efficacy in extending the shelf-life of fish products. Ekhtiarzadeh *et al.* (Ekhtiarzadeh et al., 2012) verified that V. parahaemolyticus inoculated in fish was completely inhibited by 0.75 mg/ml nisin plus 0.405% Zataria multiflora Boiss EO, 0.045% Z. multiflora Boiss EO, and 0.75 μg/ml nisin, at days 2, 6, and 9, respectively. However, *Listeria monocytogenes* was completely inhibited by EO (0.405%) plus Ni (0.25 or 0.75 mg/ml) at 1 day.

The main factors restricting the application of bacteriocins as preservatives in food are the low yield and the high cost of production (Makkar et al., 2011). One of the main ways to improve bacteriocins production is to engineer bacteriocins producing strains by increasing the copy number of the regulation and resistance genes involved in bacteriocins biosynthesis (Cheigh et al., 2005; Simsek et al., 2009). Introduction of acid tolerant genes or over-expression genes in lactic acid synthesis pathway also could improve bacteriocins production as these methods could improve the tolerance of cells to acidic conditions (Zhang et al., 2016). Increasing the carbon conversion rates in central pathway under oxidizing conditions by expressing relavant genes also could increase biomass and bacteriocins levels (Papagianni and Avramidis 2012).

#### 5.2.4 Bioactive Peptides

Bioactive peptides are specific fragments of proteins containing 2–20 amino acid residues, which can be produced by hydrolyzing proteins with a variety of proteases. Bioactive peptides have many beneficial functions, including antithrombotic, antibacterial, and antioxidant activities (Harnedy and FitzGerald 2012). And some bioactive peptides have multifunctional properties. The size, conformation, amino acid composition, and sequence of a peptide mostly affected its bioactivities (Ngo et al., 2014). According to antimicrobial properties, bioactive peptides can be divided into two main groups: those that act on the plasma membrane of the cell, and those that do not cause substantial membrane disturbance once they enter the cell (Perez Espitia et al., 2012). The key role of bioactive peptides in aquatic products as antioxidants is to prevent the formation of free radicals or to scavenge reactive oxygen species and free radicals (Irshad et al., 2015).

Hydrolysates containing peptides can be added directly to aquatic products as antioxidants or antibacterial agents. Adding 1.0 and 1.5% fish protein hydrolysate produced from yellowfin tuna waste using Protamex™ protease in minced silver carp meat extended the shelf-life of the product to 12 days compared to 6 days for the control, and resulted in retarding of lipid oxidation and microbial growth (Pezeshk et al., 2017). The addition of 2.0% grass carp protein hydrolysate in fish mince also lowered the lipid oxidation of the product (Li et al., 2015).

The bitterness of peptides caused by hydrophobic amino acids is one of the main obstacles to the application of active peptides in aquatic products (Newman et al., 2015). The bitterness can have a negative effect on the sensory properties of the product. This limitation can be addressed by various methods, such as the use of exopeptidase, plastein reaction, solvent extraction, macroporous adsorption resin, or a combination of these methods (Leksrisompong et al., 2012).

### 5.3 Biological Preservation Technology

Biological preservation is a novel and natural technology which uses microorganisms to inhibit the growth of spoilage bacteria to prolong the shelf -life of food. LAB and bacteriophages as biopreservatives have been explored to prevent fishery product spoilage.

#### 5.3.1 Lactic Acid Bacteria as Biopreservatives

Since most LABs are generally considered as safe, they have great application potential in biological preservation and naturally dominate the microflora of many foods (Ghanbari et al., 2013). Aquatic products LABs are compatible with aquatic products environments including MAP, low temperatures and pH, high salt concentration, presence of additives. Their growth can also suppress many bacteria by competing for nutrients or producing one or more metabolites with antimicrobial activity (Ghanbari et al., 2013).

Many spoilage bacteria including *Pseudomonas*, Enterobacteriaceae, and H_2_S producing bacteria (Angiolillo et al., 2018), and athogenic bacteria like V. parahaemolyticus and *L. monocytogenes* (Tahiri et al., 2009) have been efficiently inhibited by LAB. The most commonly used LAB in fishery products preservation is *Lactobacillus* spp. (Angiolillo et al., 2018), followed by *Lactococcus* spp. (Fall et al., 2010).

However, some LAB metabolites like lactic acid may influence the sensory characteristics of fishery products. These negative effects may be mitigated by combining LAB and their antibacterial metabolites with active packaging materials. Sea bass fillets coated with sodium alginate containing *L. rhamnosus* and its metabolite reuterin had a better sensory quality when compared to the control (Angiolillo et al., 2018).

#### 5.3.2 Bacteriophages as Biopreservatives

Bacteriophages are considered as promising new kinds of biopreservation agents as they can lyse target bacteria efficiently and specifically. A cocktail composed of three phages could efficiently inhibit the growth of *Shewanella* inoculated in catfish fillets, and the quality indices of phages treated samples also showed considerable improvement compared with control samples (Yang et al., 2019).

Although biopreservation strategies using LAB or bacteriophages have many advantages, such as reducing the use of chemical preservatives (Ghanbari et al., 2013), the safety and regulatory issues of LAB and phages must be seriously considered (Ben Said, Gaudreau et al., 2019).

## 6 Conclusion

The spoilage of aquatic products is mainly influenced by the composition of the microbiome, and microbial interactions need to be further explored to uncover the spoilage mechanisms. Chemical measurements are valuable and sensitive methods to evaluate the freshness of aquatic products. But their destructive testing still needs to overcome some more difficult tasks which will improve the level of chemical measurements, thus improve the speed and accuracy of aquatic products freshness evaluation. Multiple analysis techniques should be combined to determine SSOs for exploring the spoilage mechanism of SSOs. Meanwhile, biotechnologies, such as metabolomics, metagenomics and metatranscriptomics, are expected to provide a holistic view of the functional profiles of the total microflora in aquatic products, and to help analyze potential interactions between different microorganisms (Zhuang et al., 2021).

Many quality control techniques have been proved to be effective in inhibiting the growth of spoilage bacteria and extending the shelf-life of aquatic products. However, combination of different preservatives or methods will result in increased efficacy in prolonging the shelf-life of aquatic products, and developing more effective SSOs control technologies. With the development of synthetic biology, the production of existing natural preservatives such as bacteriocins will be rapidly improved, and more and more new, cheap and high-yield natural preservatives will be developed. Synthetic biology will not only promote the study of microbial spoilage mechanism of aquatic products, but also promote the development of preservation technology of aquatic products.

## References

[B1] Abdollahzadeh E. Rezaei M. Hosseini H. (2014). Antibacterial Activity of Plant Essential Oils and Extracts: The Role of Thyme Essential Oil, Nisin, and Their Combination to Control *Listeria Monocytogenes* Inoculated in Minced Fish Meat. Food Control 35 (1), 177–183. 10.1016/j.foodcont.2013.07.004

[B2] Abisado R. G. Benomar S. Klaus J. R. Dandekar A. A. Chandler J. R. (2018). Bacterial Quorum Sensing and Microbial Community Interactions. mBio 9 (3), 1–13. 10.1128/mBio.02331-17 PMC5964356 29789364

[B4] Alak G. (2012). The Effect of Chitosan Prepared in Different Solvents on the Quality Parameters of Brown Trout Fillets (*Salmo trutta* Fario). Food Nutr. Sci. 03 (09), 1303–1306. 10.4236/fns.2012.39172

[B5] Alishahi A. Aïder M. (2011). Applications of Chitosan in the Seafood Industry and Aquaculture: A Review. Food Bioproc. Technol 5 (3), 817–830. 10.1007/s11947-011-0664-x

[B6] Alparslan Y. Baygar T. (2017). Effect of Chitosan Film Coating Combined with orange Peel Essential Oil on the Shelf Life of deepwater Pink Shrimp. Food Bioproc. Technol 10 (5), 842–853. 10.1007/s11947-017-1862-y

[B7] Angiolillo L. Conte A. Del Nobile M. A. (2018). A New Method to Bio-Preserve Sea Bass Fillets. Int. J. Food Microbiol. 271, 60–66. 10.1016/j.ijfoodmicro.2018.01.010 29494893

[B8] Ashwitha A. Thamizharasan K. Vithya V. Karthik R. Bharathi S V. (2017). Effectiveness of Bacteriocin from *Bacillus Subtilis* (KY808492) and its Application in Biopreservation. fisheries-aqua 11, 36–42. 10.21767/1307-234X.1000127

[B9] Bai A. J. Rai Vittal R. (2014). Quorum Sensing Regulation and Inhibition of Exoenzyme Production and Biofilm Formation in the Food Spoilage Bacteria *Pseudomonas psychrophila* PSPF19. Food Biotechnol. 28 (4), 293–308. 10.1080/08905436.2014.963601

[B10] Bakar J. Yassoralipour A. Bakar F. A. Rahman R. A. (2010). Biogenic Amine Changes in Barramundi (*Lates calcarifer*) Slices Stored at 0°C and 4°C. Food Chem. 119 (2), 467–470. 10.1016/j.foodchem.2009.06.041

[B11] Bali V. Panesar P. S. Bera M. B. (2016). Trends in Utilization of Agro-Industrial Byproducts for Production of Bacteriocins and Their Biopreservative Applications. Crit. Rev. Biotechnol. 36 (2), 204–214. 10.3109/07388551.2014.947916 25430892

[B12] Bekaert K. Devriese L. Maes S. Robbens J. (2015). Characterization of the Dominant Bacterial Communities during Storage of Norway Lobster and Norway Lobster Tails ( *Nephrops norvegicus* ) Based on 16S rDNA Analysis by PCR-DGGE. Food Microbiol. 46, 132–138. 10.1016/j.fm.2014.06.022 25475276

[B13] Ben Said L. Gaudreau H. Dallaire L. Tessier M. Fliss I. (2019). Bioprotective Culture: a New Generation of Food Additives for the Preservation of Food Quality and Safety. Ind. Biotechnol. 15 (3), 138–147. 10.1089/ind.2019.29175.lbs

[B14] Biji K. B. Ravishankar C. N. Venkateswarlu R. Mohan C. O. Gopal T. K. S. (2016). Biogenic Amines in Seafood: a Review. J. Food Sci. Technol. 53 (5), 2210–2218. 10.1007/s13197-016-2224-x 27407186 PMC4921096

[B15] Bloch C. A. Rode C. K. (1996). Pathogenicity Island Evaluation in *Escherichia coli* K1 by Crossing with Laboratory Strain K-12. Infect. Immun. 64 (8), 3218–3223. 10.1128/iai.64.8.3218-3223.1996 8757856 PMC174210

[B16] Borrell N. Acinas S. G. Figueras M. J. Martínez-Murcia A. J. (1997). Identification of *Aeromonas* Clinical Isolates by Restriction Fragment Length Polymorphism of PCR-Amplified 16S rRNA Genes. J. Clin. Microbiol. 35 (7), 1671–1674. 10.1128/jcm.35.7.1671-1674.1997 9196171 PMC229819

[B17] Cao Y. Fanning S. Proos S. Jordan K. Srikumar S. (2017). A Review on the Applications of Next Generation Sequencing Technologies as Applied to Food-Related Microbiome Studies. Front. Microbiol. 8, 1–16. 10.3389/fmicb.2017.01829 29033905 PMC5627019

[B18] Chaillou S. Chaulot-Talmon A. Caekebeke H. Cardinal M. Christieans S. Denis C. (2015). Origin and Ecological Selection of Core and Food-specific Bacterial Communities Associated with Meat and Seafood Spoilage. ISME J. 9 (5), 1105–1118. 10.1038/ismej.2014.202 25333463 PMC4409155

[B19] Cheigh C.-I. Park H. Choi H.-J. Pyun Y.-R. (2005). Enhanced Nisin Production by Increasing Genes Involved in Nisin Z Biosynthesis in *Lactococcus Lactis* Subsp. *Lactis* A164. Biotechnol. Lett. 27 (3), 155–160. 10.1007/s10529-004-7661-3 15717123

[B20] Cheng J.-H. Sun D.-W. Zeng X.-A. Liu D. (2015). Recent Advances in Methods and Techniques for Freshness Quality Determination and Evaluation of Fish and Fish Fillets: a Review. Crit. Rev. Food Sci. Nutr. 55 (7), 1012–1225. 10.1080/10408398.2013.769934 24915394

[B21] Chong C. Y. Abu Bakar F. Russly A. R. Jamilah B. Mahyudin N. A. (2011). The Effects of Food Processing on Biogenic Amines Formation. Int. Food Res. J. 18 (3), 867–876.

[B22] Chouliara E. Karatapanis A. Savvaidis I. N. Kontominas M. G. (2007). Combined Effect of Oregano Essential Oil and Modified Atmosphere Packaging on Shelf-Life Extension of Fresh Chicken Breast Meat, Stored at 4°C. Food Microbiol. 24 (6), 607–617. 10.1016/j.fm.2006.12.005 17418312

[B23] DeWitt C. Oliveira A. (2016). Modified Atmosphere Systems and Shelf Life Extension of Fish and Fishery Products. Foods 5 (3), 48–27. 10.3390/foods5030048 PMC5302388 28231143

[B24] Di Bella J. M. Bao Y. Gloor G. B. Burton J. P. Reid G. (2013). High Throughput Sequencing Methods and Analysis for Microbiome Research. J. Microbiol. Methods 95 (3), 401–414. 10.1016/j.mimet.2013.08.011 24029734

[B25] Du W.-X. Lin C.-M. Phu A.-T. Cornell J. A. Marshall M. R. Wei C.-I. (2002). Development of Biogenic Amines in Yellowfin Tuna (*Thunnus albacares*): Effect of Storage and Correlation with Decarboxylase-Positive Bacterial flora. J. Food Sci. 67 (1), 292–301. 10.1111/j.1365-2621.2002.tb11400.x

[B26] Duan S. Zhou X. Miao J. Duan X. (2018). Succession of Bacterial Microbiota in tilapia Fillets at 4 °C and *In Situ* Investigation of Spoilers. World J. Microbiol. Biotechnol. 34 (69), 1–9. 10.1007/s11274-018-2452-5 29761232

[B27] Ekhtiarzadeh H. Akhondzadeh Basti A. Misaghi A. Sari A. Khanjari A. Rokni N. (2012). Growth Response of *Vibrio Parahaemolyticus* and *Listeria Monocytogenes* in Salted Fish Fillets as Affected by *Zataria Multiflora* Boiss. Essential Oil, Nisin, and Their Combination. J. Food Saf. 32 (3), 263–269. 10.1111/j.1745-4565.2012.00376.x

[B28] Fall P. A. Leroi F. Cardinal M. Chevalier F. Pilet M. F. (2010). Inhibition of Brochothrix Thermosphactaand Sensory Improvement of Tropical Peeled Cooked Shrimp by *Lactococcus piscium* CNCM I-4031. Lett. Appl. Microbiol. 50 (4), 357–361. 10.1111/j.1472-765X.2010.02801.x 20132434

[B29] Fernández K. Aspe E. Roeckel M. (2009). Shelf-life Extension on Fillets of Atlantic Salmon (*Salmo salar*) Using Natural Additives, Superchilling and Modified Atmosphere Packaging. Food Control 20 (11), 1036–1042. 10.1016/j.foodcont.2008.12.010

[B30] Figueras M. J. Soler L. Chacón M. R. Guarro J. Martínez-Murcia A. J. (2000). Extended Method for Discrimination of *Aeromonas* Spp. By 16S rDNA RFLP Analysis. Int. J. Syst. Evol. Microbiol. 50 (6), 2069–2073. 10.1099/00207713-50-6-2069 11155981

[B31] Fischer S. G. Lerman L. S. (1983). DNA Fragments Differing by Single Base-Pair Substitutions Are Separated in Denaturing Gradient Gels: Correspondence with Melting Theory. Proc. Natl. Acad. Sci. U.S.A. 80 (6), 1579–1583. 10.1073/pnas.80.6.1579 6220406 PMC393645

[B32] Flórez A. B. Mayo B. (2006). PCR-DGGE as a Tool for Characterizing Dominant Microbial Populations in the Spanish Blue-Veined Cabrales Cheese. Int. Dairy J. 16 (10), 1205–1210. 10.1016/j.idairyj.2005.11.008

[B33] Fu L. Wang C. Liu N. Ma A. Wang Y. (2018). Quorum sensing System-Regulated Genes Affect the Spoilage Potential of *Shewanella Baltica* . Food Res. Int. 107, 1–9. 10.1016/j.foodres.2018.01.067 29580465

[B34] Fuqua W. C. Winans S. C. Greenberg E. P. (1994). Quorum sensing in Bacteria: the LuxR-LuxI Family of Cell Density-Responsive Transcriptional Regulators. J. Bacteriol. 176 (2), 269–275. 10.1128/jb.176.2.269-275.1994 8288518 PMC205046

[B35] Gallart-Jornet L. Rustad T. Barat J. M. Fito P. Escriche I. (2007). Effect of Superchilled Storage on the Freshness and Salting Behaviour of Atlantic salmon (*Salmo salar*) Fillets. Food Chem. 103 (4), 1268–1281. 10.1016/j.foodchem.2006.10.040

[B36] García-Díez J. Alheiro J. Pinto A. L. Soares L. Falco V. Fraqueza M. J. (2016). Behaviour of Food-Borne Pathogens on Dry Cured Sausage Manufactured with Herbs and Spices Essential Oils and Their Sensorial Acceptability. Food Control 59, 262–270. 10.1016/j.foodcont.2015.05.027

[B37] Ge C. Lee C. S. Yu Z. Lee J. (2012). Comparison of Bacterial Profiles of Fish between Storage Conditions at Retails Using DGGE and Banding Pattern Analysis: Consumer's Perspective. Food Nutr. Sci. 03 (02), 190–200. 10.4236/fns.2012.32028

[B38] Ghanbari M. Jami M. Domig K. J. Kneifel W. (2013). Seafood Biopreservation by Lactic Acid Bacteria - A Review. LWT - Food Sci. Tech. 54 (2), 315–324. 10.1016/j.lwt.2013.05.039

[B39] Gu Q. Fu L. Wang Y. Lin J. (2013). Identification and Characterization of Extracellular Cyclic Dipeptides as Quorum-Sensing Signal Molecules fromShewanella Baltica, the Specific Spoilage Organism ofPseudosciaena Croceaduring 4 °C Storage. J. Agric. Food Chem. 61 (47), 11645–11652. 10.1021/jf403918x 24206027

[B40] Gudipati V. (2017). Role of Plant Extracts as Natural Additives in Fish and Fish Products - A Review. Fish. Tech. 54, 145–154.

[B41] Gui M. Liu L. Wu R. Hu J. Wang S. Li P. (2018). Detection of New Quorum Sensing N-Acyl Homoserine Lactones from Aeromonas Veronii. Front. Microbiol. 9, 1–9. 10.3389/fmicb.2018.01712 30108567 PMC6079219

[B42] Harnedy P. A. FitzGerald R. J. (2012). Bioactive Peptides from marine Processing Waste and Shellfish: A Review. J. Funct. Foods 4 (1), 6–24. 10.1016/j.jff.2011.09.001

[B43] Hauptmann A. L. Paulová P. Castro-Mejía J. L. Hansen L. H. Sicheritz-Pontén T. Mulvad G. (2020). The Microbial Composition of Dried Fish Prepared According to Greenlandic Inuit Traditions and Industrial Counterparts. Food Microbiol. 85, 103305–103311. 10.1016/j.fm.2019.103305 31500717

[B44] Hovda M. B. Fontanillas R. McGurk C. Obach A. Rosnes J. T. (2012). Seasonal Variations in the Intestinal Microbiota of Farmed Atlantic salmon (*Salmo salar* L.). Aquac. Res. 43 (1), 154–159. 10.1111/j.1365-2109.2011.02805.x

[B45] Hovda M. B. Lunestad B. T. Sivertsvik M. Rosnes J. T. (2007a). Characterisation of the Bacterial flora of Modified Atmosphere Packaged Farmed Atlantic Cod (*Gadus morhua*) by PCR-DGGE of Conserved 16S rRNA Gene Regions. Int. J. Food Microbiol. 117 (1), 68–75. 10.1016/j.ijfoodmicro.2007.02.022 17467836

[B46] Hovda M. B. Sivertsvik M. Tore Lunestad B. Lorentzen G. Rosnes J. T. (2007b). Characterisation of the Dominant Bacterial Population in Modified Atmosphere Packaged Farmed Halibut (*Hippoglossus hippoglossus*) Based on 16S rDNA-DGGE. Food Microbiol. 24 (4), 362–371. 10.1016/j.fm.2006.07.018 17189762

[B47] Hwang C.-C. Lee Y.-C. Huang Y.-R. Lin C.-M. Shiau C.-Y. Hwang D.-F. (2010). Biogenic Amines Content, Histamine-Forming Bacteria and Adulteration of Bonito in Tuna Candy Products. Food Control 21 (6), 845–850. 10.1016/j.foodcont.2009.11.011

[B48] Irshad I. Kanekanian A. Peters A. Masud T. (2015). Antioxidant Activity of Bioactive Peptides Derived from Bovine Casein Hydrolysate Fractions. J. Food Sci. Technol. 52 (1), 231–239. 10.1007/s13197-012-0920-8

[B49] Jia S. Li Y. Zhuang S. Sun X. Zhang L. Shi J. (2019). Biochemical Changes Induced by Dominant Bacteria in Chill-Stored Silver Carp (*Hypophthalmichthys molitrix*) and GC-IMS Identification of Volatile Organic Compounds. Food Microbiol. 84, 1–11. 10.1016/j.fm.2019.103248 31421785

[B50] Leksrisompong P. Gerard P. Lopetcharat K. Drake M. (2012). Bitter Taste Inhibiting Agents for Whey Protein Hydrolysate and Whey Protein Hydrolysate Beverages. J. Food Sci. 77 (8), S282–S287. 10.1111/j.1750-3841.2012.02800.x 22809256

[B51] Leroi F. Cornet J. Chevalier F. Cardinal M. Coeuret G. Chaillou S. (2015). Selection of Bioprotective Cultures for Preventing Cold-Smoked salmon Spoilage. Int. J. Food Microbiol. 213, 79–87. 10.1016/j.ijfoodmicro.2015.05.005 26044337

[B52] Li Liu L. Yan Y. Feng L. Zhu J. (2018). Quorum sensing *asaI* Mutants Affect Spoilage Phenotypes, Motility, and Biofilm Formation in a marine Fish Isolate of *Aeromonas Salmonicida* . Food Microbiol. 76, 40–51. 10.1016/j.fm.2018.04.009 30166167

[B53] Li X. Luo Y. You J. Shen H. (2015). Stability of Papain-Treated Grass Carp (*Ctenopharyngodon idellus*) Protein Hydrolysate during Food Processing and its Ability to Inhibit Lipid Oxidation in Frozen Fish Mince. J. Food Sci. Technol. 52 (1), 542–548. 10.1007/s13197-013-1031-x

[B54] Li T. Cui F. Bai F. Zhao G. Li J. (2016). Involvement of Acylated Homoserine Lactones (AHLs) of *Aeromonas Sobria* in Spoilage of Refrigerated Turbot (*Scophthalmus maximus* L.). Sensors 16 (7), 1–14. 10.3390/s16071083 PMC4970129 27420072

[B3] Liu A. F. Xie J. Qian Y. F. (2018). Spoilage Potential of Dominant Spoilage Bacteria from Chilled Tuna (*Thunnus Obesus*). Chin. Food Sci 39 (3), 7–14. 10.7506/spkx1002-6630-201803002

[B55] Lodhi G. Kim Y.-S. Hwang J.-W. Kim S.-K. Jeon Y.-J. Je J.-Y. (2014). Chitooligosaccharide and its Derivatives: Preparation and Biological Applications. Biomed. Res. Int. 2014, 1–13. 10.1155/2014/654913 PMC3958764 24724091

[B56] López‐Porras A. Griffin M. J. Armwood A. R. Camus A. C. Waldbieser G. C. Ware C. (2021). Genetic Variability of Edwardsiella Piscicida Isolates from Mississippi Catfish Aquaculture with an Assessment of Virulence in Channel and Channel × Blue Hybrid Catfish. J. Fish. Dis. 44 (11), 1725–1751. 10.1111/jfd.13491 34251059

[B57] Ma Y. Sun X. Zhao Y. Lu Y. Vivian C. H. Pan Y. (2013). REP-PCR and ERIC-PCR Analysis for the Typing of *Vibrio Parahaemolyticus* Isolated from Sea Products Marketed in Shanghai. Chin. Food Sci. 34(10): 263–267. 10.7506/spkx1002-6630-201310058

[B58] Machado I. Silva L. R. Giaouris E. D. Melo L. F. Simões M. (2020). Quorum sensing in Food Spoilage and Natural-Based Strategies for its Inhibition. Food Res. Int. 127, 1–12. 10.1016/j.foodres.2019.108754 31882100

[B59] Magnussen O. M. Haugland A. Torstveit Hemmingsen A. K. Johansen S. Nordtvedt T. S. (2008). Advances in Superchilling of Food - Process Characteristics and Product Quality. Trends Food Sci. Tech. 19 (8), 418–424. 10.1016/j.tifs.2008.04.005

[B60] Makkar R. S. Cameotra S. S. Banat I. M. (2011). Advances in Utilization of Renewable Substrates for Biosurfactant Production. AMB Express. 1(5): 5–19. 10.1186/2191-0855-1-5 21906330 PMC3159906

[B61] Maqsood S. Benjakul S. Abushelaibi A. Alam A. (2014). Phenolic Compounds and Plant Phenolic Extracts as Natural Antioxidants in Prevention of Lipid Oxidation in Seafood: A Detailed Review. Compr. Rev. Food Sci. Food Saf. 13 (6), 1125–1140. 10.1111/1541-4337.12106

[B62] Martínez-Alvarez O. Gómez-Guillén C. (2013). Influence of Mono- and Divalent Salts on Water Loss and Properties of Dry Salted Cod Fillets. LWT - Food Sci. Tech. 53 (2), 387–394. 10.1016/j.lwt.2013.04.013

[B63] McCormack J. (2006). Quorum sensing, Bacterial Communication and New Antibiotics. Intern. Med. J. 36 (12), 757–758. 10.1111/j.1445-5994.2006.01232.x 17096737

[B64] Mohan C. O. Ravishankar C. N. Lalitha K. V. Srinivasa Gopal T. K. (2012). Effect of Chitosan Edible Coating on the Quality of Double Filleted Indian Oil Sardine (*Sardinella Longiceps*) during Chilled Storage. Food Hydrocolloids 26 (1), 167–174. 10.1016/j.foodhyd.2011.05.005

[B65] Muyzer G. de Waal E. C. Uitterlinden A. G. (1993). Profiling of Complex Microbial Populations by Denaturing Gradient Gel Electrophoresis Analysis of Polymerase Chain Reaction-Amplified Genes Coding for 16S rRNA. Appl. Environ. Microbiol. 59 (3), 695–700. 10.1128/aem.59.3.695-70010.1128/aem.59.3.695-700.1993 7683183 PMC202176

[B66] Navarro‐Segura L. Ros‐Chumillas M. Martínez‐Hernández G. B. López‐Gómez A. (2020). A New Advanced Packaging System for Extending the Shelf Life of Refrigerated Farmed Fish Fillets. J. Sci. Food Agric. 100 (12), 4601–4611. 10.1002/jsfa.10520 32419139

[B67] Newman J. O'Riordan D. Jacquier J. C. O'Sullivan M. (2015). Masking of Bitterness in Dairy Protein Hydrolysates: Comparison of an Electronic Tongue and a Trained Sensory Panel as Means of Directing the Masking Strategy. LWT - Food Sci. Tech. 63 (1), 751–757. 10.1016/j.lwt.2015.03.019

[B68] Ngo D.-H. Ryu B. Kim S.-K. (2014). Active Peptides from Skate (*Okamejei kenojei*) Skin Gelatin Diminish Angiotensin-I Converting Enzyme Activity and Intracellular Free Radical-Mediated Oxidation. Food Chem. 143, 246–255. 10.1016/j.foodchem.2013.07.067 24054237

[B69] No H. K. Meyers S. P. Prinyawiwatkul W. Xu Z. (2007). Applications of Chitosan for Improvement of Quality and Shelf Life of Foods: a Review. J. Food Sci. 72 (5), R87–R100. 10.1111/j.1750-3841.2007.00383.x 17995743

[B70] Ocaño-Higuera V. M. Maeda-Martínez A. N. Marquez-Ríos E. Canizales-Rodríguez D. F. Castillo-Yáñez F. J. Ruíz-Bustos E. (2011). Freshness Assessment of ray Fish Stored in Ice by Biochemical, Chemical and Physical Methods. Food Chem. 125 (1), 49–54. 10.1016/j.foodchem.2010.08.034

[B71] Olatunde O. O. Benjakul S. (2018). Natural Preservatives for Extending the Shelf-Life of Seafood: A Revisit. Compr. Rev. Food Sci. Food Saf. 17 (6), 1595–1612. 10.1111/1541-4337.12390 33350137

[B72] Olatunde O. O. Benjakul S. Vongkamjan K. (2020). Shelf-life of Refrigerated Asian Sea Bass Slices Treated with Cold Plasma as Affected by Gas Composition in Packaging. Int. J. Food Microbiol. 324, 1–11. 10.1016/j.ijfoodmicro.2020.108612 32244103

[B73] Önal A. Tekkeli S. E. K. Önal C. (2013). A Review of the Liquid Chromatographic Methods for the Determination of Biogenic Amines in Foods. Food Chem. 138 (1), 509–515. 10.1016/j.foodchem.2012.10.056 23265518

[B74] Ormanci H. B. Colakoglu F. A. (2015). Nutritional and Sensory Properties of Salted Fish Product, Lakerda. Cogent Food Agric. 1 (1), 1–13. 10.1080/23311932.2015.1008348

[B75] Özogul F. Özogul Y. (2006). Biogenic Amine Content and Biogenic Amine Quality Indices of Sardines (*Sardina Pilchardus*) Stored in Modified Atmosphere Packaging and Vacuum Packaging. Food Chem. 99 (3), 574–578. 10.1016/j.foodchem.2005.08.029

[B76] Ozogul Y. Ozyurt G. Ozogul F. Kuley E. Polat A. (2005). Freshness Assessment of European eel (*Anguilla anguilla*) by Sensory, Chemical and Microbiological Methods. Food Chem. 92 (4), 745–751. 10.1016/j.foodchem.2004.08.035

[B77] Pan Z. Chen Y. Li B. Hu S. Yang Y. Li L. (2013). Specifi C Spoilage Organism of Vacuum-Packed Crisped Grass Carp during Chilled Storage. Food Sci. Tech. 38 (09), 109–113. 10.13684/j.cnki.spkj.2013.09.055

[B78] Papagianni M. Avramidis N. (2012). Engineering the central Pathways in *Lactococcus Lactis*: Functional Expression of the Phosphofructokinase (*Pfk*) and Alternative Oxidase (*Aox1*) Genes from *Aspergillus niger* in *Lactococcus Lactis* Facilitates Improved Carbon Conversion Rates under Oxidizing Conditions. Enzyme Microb. Tech. 51 (3), 125–130. 10.1016/j.enzmictec.2012.04.007 22759530

[B79] Parlapani F. F. Michailidou S. Anagnostopoulos D. A. Koromilas S. Kios K. Pasentsis K. (2019). Bacterial Communities and Potential Spoilage Markers of Whole Blue Crab (*Callinectes sapidus*) Stored under Commercial Simulated Conditions. Food Microbiol. 82, 325–333. 10.1016/j.fm.2019.03.011 31027790

[B80] Parlapani F. F. Ferrocino I. Michailidou S. Argiriou A. Haroutounian S. A. Kokokiris L. (2020). Microbiota and Volatilome Profile of Fresh and Chill-Stored deepwater Rose Shrimp (*Parapenaeus Longirostris*). Food Res. Int. 132, 109057–109058. 10.1016/j.foodres.2020.109057 32331667

[B81] Paydar M. Teh C. S. J. Thong K. L. (2013). Prevalence and Characterisation of Potentially Virulent *Vibrio Parahaemolyticus* in Seafood in Malaysia Using Conventional Methods, PCR and REP-PCR. Food Control 32 (1), 13–18. 10.1016/j.foodcont.2012.11.034

[B82] Perez Espitia P. J. de Fátima Ferreira Soares N. Dos Reis Coimbra J. S. de Andrade N. J. Souza Cruz R. Alves Medeiros E. A. (2012). Bioactive Peptides: Synthesis, Properties, and Applications in the Packaging and Preservation of Food. Compr. Rev. Food Sci. Food Saf. 11 (2), 187–204. 10.1111/j.1541-4337.2011.00179.x 32368201 PMC7194098

[B83] Perez R. Perez M. T. Elegado F. (2015). Bacteriocins from Lactic Acid Bacteria: A Review of Biosynthesis, Mode of Action, Fermentative Production, Uses, and Prospects. PhilSciTech 8 (2), 61–67. 10.18191/2015-08-2-027

[B84] Pezeshk S. Ojagh S. M. Rezaei M. Shabanpour B. (2017). Antioxidant and Antibacterial Effect of Protein Hydrolysis of Yellowfin Tuna Waste on Flesh Quality Parameters of Minced Silver Carp. J. Genet. Res 3 (2), 103–112. 10.22080/jgr.2018.13611.1091

[B85] Pikuta E. V. Hoover R. B. Tang J. (2007). Microbial Extremophiles at the Limits of Life. Crit. Rev. Microbiol. 33 (3), 183–209. 10.1080/10408410701451948 17653987

[B86] Pons-Sánchez-Cascado S. Veciana-Nogués M. T. Bover-Cid S. Mariné-Font A. Vidal-Carou M. C. (2006). Use of Volatile and Non-volatile Amines to Evaluate the Freshness of Anchovies Stored in Ice. J. Sci. Food Agric. 86 (5), 699–705. 10.1002/jsfa.2398

[B87] Que T. Liu W. Chen S. Liu D. Ye X. Hu Y. (2013). Research Progress of Low Temperature Preservation Technology in Aquatic Products. J. Chin. Inst Food Sci Tech 13 (8), 181–189. 10.16429/j.1009-7848.2013.08.003

[B88] Que T. Zheng J. Chen S. iang Q. Liu W. Ye X. (2015). Effect of Super-chilling and Frozen on the Meat Quality of Snakehead. J. Chin. Inst Food Sci Tech 15 (6), 136–147. 10.16429/j.1009-7848.2015.06.019

[B89] Rahman M. Somsiri T. Tanaka R. Sawabe T. Tajima K. (2005). PCR-RFLP Analysis for Identification of *Aeromonas* Isolates Collected from Diseased Fish and Aquatic Animals. Fish. Pathol. 40 (4), 151–159. 10.3147/jsfp.40.151

[B90] Reading N. C. Sperandio V. (2006). Quorum sensing: the many Languages of Bacteria. FEMS Microbiol. Lett. 254 (1), 1–11. 10.1111/j.1574-6968.2005.00001.x 16451172

[B91] Rodriguez-Casado A. Carmona P. Moreno P. Sánchez-González I. Macagnano A. Natale C. D. (2007). Structural Changes in Sardine (*Sardina Pilchardus*) Muscle during Iced Storage: Investigation by DRIFT Spectroscopy. Food Chem. 103 (3), 1024–1030. 10.1016/j.foodchem.2006.09.054

[B92] Rong C. Qi L. Bang-zhong Y. Lan-lan Z. (2010). Combined Effect of Ozonated Water and Chitosan on the Shelf-Life of Pacific Oyster (*Crassostrea gigas*). Innovative Food Sci. Emerging Tech. 11 (1), 108–112. 10.1016/j.ifset.2009.08.006

[B93] Rong C. Ling Z. Huihui S. Qi L. (2018). Characterization of Microbial Community in High-Pressure Treated Oysters by High-Throughput Sequencing Technology. Innovative Food Sci. Emerging Tech. 45, 241–248. 10.1016/j.ifset.2017.11.001

[B94] Schwenteit J. Gram L. Nielsen K. F. Fridjonsson O. H. Bornscheuer U. T. Givskov M. (2011). Quorum sensing in *Aeromonas Salmonicida* Subsp. *Achromogenes* and the Effect of the Autoinducer Synthase AsaI on Bacterial Virulence. Vet. Microbiol. 147 (3-4), 389–397. 10.1016/j.vetmic.2010.07.020 20708354

[B95] Sheffield V. C. Cox D. R. Lerman L. S. Myers R. M. (1989). Attachment of a 40-Base-Pair G + C-Rich Sequence (GC-Clamp) to Genomic DNA Fragments by the Polymerase Chain Reaction Results in Improved Detection of Single-Base Changes. Proc. Natl. Acad. Sci. U.S.A. 86 (1), 232–236. 10.1073/pnas.86.1.232 2643100 PMC286438

[B96] Shouchun L. Wen F. Saiyi Z. Changwei M. Pinglan L. Kang Z. (2010). Quality Evaluation of Tray-Packed tilapia Fillets Stored at 0C Based on Sensory, Microbiological, Biochemical and Physical Attributes. Afr. J. Biotechnol. 9 (5), 692–701. 10.5897/AJB09.1369

[B97] Silva-Angulo A. B. Zanini S. F. Rosenthal A. Rodrigo D. Klein G. Martínez A. (2015). Combined Effect of Carvacrol and Citral on the Growth of *Listeria Monocytogenes* and *Listeria Innocua* and on the Occurrence of Damaged Cells. Food Control 53, 156–162. 10.1016/j.foodcont.2015.01.028

[B98] Simões M. Bennett R. N. Rosa E. A. S. (2009). Understanding Antimicrobial Activities of Phytochemicals against Multidrug Resistant Bacteria and Biofilms. Nat. Prod. Rep. 26 (6), 746–757. 10.1039/b821648g 19471683

[B99] Şimşek Ö. Çon A. H. Akkoç N. Saris P. E. J. Akçelik M. (2009). Influence of Growth Conditions on the Nisin Production of Bioengineered *Lactococcus Lactis* Strains. J. Ind. Microbiol. Biotechnol. 36 (4), 481–490. 10.1007/s10295-008-0517-4 19137338

[B100] Sivertsvik M. Rosnes J. T. Kleiberg G. H. (2003). Effect of Modified Atmosphere Packaging and Superchilled Storage on the Microbial and Sensory Quality of Atlantic Salmon (*Salmo salar*) Fillets. J. Food Sci. 68, 1467–1472. 10.1111/j.1365-2621.2003.tb09668.x

[B101] Sobieszczańska N. Myszka K. Szwengiel A. Majcher M. Grygier A. Wolko Ł. (2020). Tarragon Essential Oil as a Source of Bioactive Compounds with Anti-*Quorum Sensing* and Anti-proteolytic Activity against *Pseudomonas* Spp. Isolated from Fish - *In Vitro, In Silico* and *In Situ* Approaches. Int. J. Food Microbiol. 331, 1–16. 10.1016/j.ijfoodmicro.2020.108732 32521374

[B102] Sotelo C. G. Gallardo J. M. Piñeiro C. Pérez-Martin R. (1995). Trimethylamine Oxide and Derived Compounds' Changes during Frozen Storage of Hake (*Merluccius merluccius*). Food Chem. 53, 61–65. 10.1016/0308-8146(95)95787-7

[B103] Svanevik C. S. Lunestad B. T. (2011). Characterisation of the Microbiota of Atlantic Mackerel (*Scomber scombrus*). Int. J. Food Microbiol. 151 (2), 164–170. 10.1016/j.ijfoodmicro.2011.08.016 21914558

[B104] Tahiri I. Desbiens M. Kheadr E. Lacroix C. Fliss I. (2009). Comparison of Different Application Strategies of Divergicin M35 for Inactivation of *Listeria Monocytogenes* in Cold-Smoked Wild salmon. Food Microbiol. 26 (8), 783–793. 10.1016/j.fm.2009.05.003 19835762

[B105] Taliadourou D. Papadopoulos V. Domvridou E. Savvaidis I. N. Kontominas M. G. (2003). Microbiological, Chemical and Sensory Changes of Whole and Filleted Mediterranean Aquacultured Sea Bass (*Dicentrarchus labrax*) Stored in Ice. J. Sci. Food Agric. 83 (13), 1373–1379. 10.1002/jsfa.1553

[B106] Tang R. Zhu J. Feng L. Li J. Liu X. (2019). Characterization of LuxI/LuxR and Their Regulation Involved in Biofilm Formation and Stress Resistance in Fish Spoilers *Pseudomonas Fluorescens* . Int. J. Food Microbiol. 297, 60–71. 10.1016/j.ijfoodmicro.2018.12.011 30884254

[B107] ten Brink B. Damink C. Joosten H. M. L. J. Huis in 't Veld J. H. J. (1990). Occurrence and Formation of Biologically Active Amines in Foods. Int. J. Food Microbiol. 11 (1), 73–84. 10.1016/0168-1605(90)90040-c 2223522

[B108] Trocino A. Xiccato G. Majolini D. Tazzoli M. Bertotto D. Pascoli F. (2012). Assessing the Quality of Organic and Conventionally-Farmed European Sea Bass (*Dicentrarchus labrax*). Food Chem. 131 (2), 427–433. 10.1016/j.foodchem.2011.08.082

[B109] Waters C. M. Bassler B. L. (2005). Quorum sensing: Cell-To-Cell Communication in Bacteria. Annu. Rev. Cel Dev. Biol. 21, 319–346. 10.1146/annurev.cellbio.21.012704.131001 16212498

[B110] Weerakkody N. S. Caffin N. Turner M. S. Dykes G. A. (2010). *In Vitro* antimicrobial Activity of Less-Utilized Spice and Herb Extracts against Selected Food-Borne Bacteria. Food Control 21 (10), 1408–1414. 10.1016/j.foodcont.2010.04.014

[B111] Wills C. C. Proctor M. R. M. McLoughlin J. V. (2004). Integrated Studies on the Freshness of Rainbow trout (*Oncorhynchus mykiss Walbaum*) Postmortem during Chilled and Frozen Storage. J. Food Biochem. 28 (3), 213–244. 10.1111/j.1745-4514.2004.tb00067.x

[B112] Xie J. Zhang Z. Yang S.-P. Cheng Y. Qian Y.-F. (2018). Study on the Spoilage Potential of *Pseudomonas Fluorescens* on salmon Stored at Different Temperatures. J. Food Sci. Technol. 55 (1), 217–225. 10.1007/s13197-017-2916-x 29358813 PMC5756204

[B113] Yang Z.-q. Tao X.-y. Zhang H. Rao S.-q. Gao L. Pan Z.-m. (2019). Isolation and Characterization of Virulent Phages Infecting *Shewanella Baltica* and *Shewanella Putrefaciens*, and Their Application for Biopreservation of Chilled Channel Catfish (*Ictalurus punctatus*). Int. J. Food Microbiol. 292, 107–117. 10.1016/j.ijfoodmicro.2018.12.020 30594742

[B114] Yew C. C. Bakar F. A. Rahman R. A. Bakar J. Zaman M. Z. Velu S. (2014). Effects of Modified Atmosphere Packaging with Various Carbon Dioxide Composition on Biogenic Amines Formation in Indian Mackerel (*Rastrelliger kanagurta*) Stored at 5 ± 1°C. Packag. Technol. Sci. 27 (3), 249–254. 10.1002/pts.2020

[B115] Zacharof M. P. Lovitt R. W. (2012). Bacteriocins Produced by Lactic Acid Bacteria a Review Article. APCBEE Proced. 2, 50–56. 10.1016/j.apcbee.2012.06.010

[B116] Zhang L. Murphy P. J. Kerr A. Tate M. E. (1993). Agrobacterium Conjugation and Gene Regulation by N-Acyl-L-Homoserine Lactones. Nature 362 (6419), 446–448. 10.1038/362446a0 8464475

[B117] Zhang J. Caiyin Q. Feng W. Zhao X. Qiao B. Zhao G. (2016). Enhance Nisin Yield via Improving Acid-Tolerant Capability of *Lactococcus Lactis* F44. Sci. Rep. 6, 1–12. 10.1038/srep27973 27306587 PMC4910042

[B118] Zhang J. Li Y. Liu X. Lei Y. Regenstein J. M. Luo Y. (2019). Characterization of the Microbial Composition and Quality of Lightly Salted Grass Carp (*Ctenopharyngodon idellus*) Fillets with Vacuum or Modified Atmosphere Packaging. Int. J. Food Microbiol. 293, 87–93. 10.1016/j.ijfoodmicro.2018.12.022 30677560

[B119] Zheng R. Xu X. Xing J. Cheng H. Zhang S. Shen J. (2020). Quality Evaluation and Characterization of Specific Spoilage Organisms of Spanish Mackerel by High-Throughput Sequencing during 0 °C Cold Chain Logistics. Foods 9 (3), 312–316. 10.3390/foods9030312 PMC7143841 32182816

[B120] Zhu S. Wu H. Zeng M. Liu Z. Wang Y. (2015). The Involvement of Bacterial Quorum Sensing in the Spoilage of Refrigerated *Litopenaeus Vannamei* . Int. J. Food Microbiol. 192, 26–33. 10.1016/j.ijfoodmicro.2014.09.029 25305441

[B121] Zhu J. Zhao A. Feng L. Gao H. (2016). Quorum sensing Signals Affect Spoilage of Refrigerated Large Yellow Croaker (*Pseudosciaena Crocea*) by *Shewanella Baltica* . Int. J. Food Microbiol. 217, 146–155. 10.1016/j.ijfoodmicro.2015.10.020 26519730

[B122] Zhu J. Zhang Y. Deng J. Jiang H. Zhuang L. Ye W. (2019). Diketopiperazines Synthesis Gene in *Shewanella Baltica* and Roles of Diketopiperazines and Resveratrol in Quorum Sensing. J. Agric. Food Chem. 67 (43), 12013–12025. 10.1021/acs.jafc.9b04620 31589428

[B123] Zhuang S. Hong H. Zhang L. Luo Y. (2021). Spoilage‐related Microbiota in Fish and Crustaceans during Storage: Research Progress and Future Trends. Compr. Rev. Food Sci. Food Saf. 20 (1), 252–288. 10.1111/1541-4337.12659 33443810

[B124] Zhuang S. Li Y. Jia S. Hong H. Liu Y. Luo Y. (2019). Effects of Pomegranate Peel Extract on Quality and Microbiota Composition of Bighead Carp (*Aristichthys nobilis*) Fillets during Chilled Storage. Food Microbiol. 82, 445–454. 10.1016/j.fm.2019.03.019 31027804

